# Phonological Representations Are Unconsciously Used when Processing Complex, Non-Speech Signals

**DOI:** 10.1371/journal.pone.0001966

**Published:** 2008-04-16

**Authors:** Mahan Azadpour, Evan Balaban

**Affiliations:** 1 Cognitive Neuroscience Sector, SISSA (International School for Advanced Studies), Trieste, Italy; 2 Behavioral Neurosciences Program, McGill University, Montreal, Canada; Indiana University, United States of America

## Abstract

Neuroimaging studies of speech processing increasingly rely on artificial speech-like sounds whose perceptual status as speech or non-speech is assigned by simple subjective judgments; brain activation patterns are interpreted according to these status assignments. The naïve perceptual status of one such stimulus, spectrally-rotated speech (not consciously perceived as speech by naïve subjects), was evaluated in discrimination and forced identification experiments. Discrimination of variation in spectrally-rotated syllables in one group of naïve subjects was strongly related to the pattern of similarities in phonological identification of the same stimuli provided by a second, independent group of naïve subjects, suggesting either that (1) naïve rotated syllable perception involves phonetic-like processing, or (2) that perception is solely based on physical acoustic similarity, and similar sounds are provided with similar phonetic identities. Analysis of acoustic (Euclidean distances of center frequency values of formants) and phonetic similarities in the perception of the vowel portions of the rotated syllables revealed that discrimination was significantly and independently influenced by both acoustic and phonological information. We conclude that simple subjective assessments of artificial speech-like sounds can be misleading, as perception of such sounds may initially and unconsciously utilize speech-like, phonological processing.

## Introduction

Recent behavioral and neuroimaging studies comparing speech and non-speech sound processing in the human brain have relied heavily on digitally-manipulated or synthesized sounds with speech-like acoustical properties that are not subjectively reported to be “speech” by listeners [1]–[7]. Such stimuli do not appear to be initially processed by the phonological system, but can acquire phonological properties as a result of exposure and/or training [4], [8], [9]. However, detailed studies of the initial perceptual properties of these stimuli are lacking, especially with respect to the important question of the extent to which such sounds may be unconsciously perceived in a speech-like manner. The assumption of distinct speech and non-speech modes in the initial perception of such sounds (as reflected by conscious subjective reports) is evaluated here using putatively non-speech stimuli played to naïve listeners.

**Figure 1:**
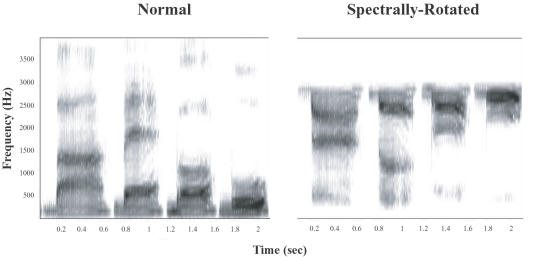
Sound spectrograms of the normal (left) and spectrally-rotated (right, 1.5 kHz rotation frequency) syllable sequence *\ba\-\be\-\bo\-\bu\* spoken by a male Italian speaker.

This study uses spectrally-rotated speech [8] (Figure 1; recorded examples provided in Supporting Information Audio S1, Audio S2), obtained by inverting the speech spectrum around a center frequency, a manipulation that has been used as a non-phonological control stimulus in human neuroimaging studies [1]–[3], [6], [7]. People can learn to perceive spectrally-rotated syllables, words and sentences as speech, but only after some hours of training [8], [9]. As part of a larger project examining how people learn to fluently perceive spectrally-rotated speech as a result of training, we first wanted to document the perceptual starting point before learning has taken place; that is, how rotated speech is naively perceived.

Spectrally-rotated speech syllables were produced by flipping the spectrum of speech around a center frequency of 1.5 kHz by applying a custom digital implementation of the original algorithm [8] to 75 Italian syllables (15 consonants, and the 5 cardinal vowels /a/,/e/,/i/,/o/,/u/ with the open and close forms of both /e/ and /o/ merged, see below) recorded from a male native speaker. This rotation frequency was chosen based on pilot work indicating that listeners who had experience with rotated speech, and those naïve to it, were less likely to describe sounds rotated about this frequency as “speech-like” than sounds produced with higher rotation frequencies used in previous studies [1]–[3], [6], [7], [8] (see Materials and Methods, Supporting Information Text S1, Tables S1, S2, S3, S4). The spectral rotation operation inverts spectral information while preserving the general form of the amplitude envelope and the temporal relationships of spectral trajectories. The perception of rotated sounds by naive listeners was studied by comparing the performance of independent groups of naïve subjects on sound discrimination and forced phonological identification tasks.

First, perceptual distances of rotated syllables from each other were constructed using data from a same/different discrimination task performed by an initial group of native-speaking Italian listeners with no prior experience with rotated speech. Subjects were presented with three rotated syllables in a row, and were instructed to indicate whether the third sound was similar to any of the other two. The number of false “same” judgments given to stimulus pairs that differed in the identity of a single consonant or vowel was used as a metric of perceptual distance, on the assumption that pairs of stimuli perceived to be more similar to each other should have a higher incidence of erroneous “same” judgments.

Data on the phonological distances of rotated sounds was then collected by compelling a second, independent set of native-speaking Italian listeners (with no previous exposure to rotated stimuli) to provide phonological descriptions of the same spectrally-rotated syllables used in the discrimination experiment. Participants typed onomatopoetic descriptions of rotated syllables on a computer keyboard (in Italian, unlike English, there is good correspondence between phonological patterns for vowels and consonants for written and spoken forms). Using these primary data, identification matrices were compiled for both rotated consonants and vowels, which represent the proportion of cases in which each rotated vowel or consonant (a row of the matrix) is identified with each unrotated vowel or consonant (the columns of the matrix). Phonological distances were quantified using the numbers in the identification matrix row values of two rotated sounds, on the assumption that sounds that are more phonologically distant should exhibit a more divergent pattern of identification values across vowel and consonant categories.

By using stimuli that none of the subjects reported hearing as speech, and comparing phonological distance with perceptual distance data collected from two independent, naïve groups of subjects, we could assure that any pattern of resemblances between these two data sets reflects the naïve perceptual status of the stimuli. If the pattern of perceptual differences significantly resembles the pattern of phonological distances, there are two possible explanations: either variation in rotated syllables is naïvely perceived in a phonetic-like manner to some extent, or perception is solely based on physical acoustic similarity, and two sounds that are acoustically similar will be given similar phonological descriptions. These explanations can be tested by quantifying the acoustic distance relations among stimulus sounds and examining whether phonological and acoustic distances independently account for variation in discrimination performance.

In order to be able to measure the physical acoustic similarities between sounds in a perceptually-valid, uniform and reliable way, we focused on a subset of the information in the stimulus sounds, the vowels. Vowels were selected because consonants vary widely in their meaningful acoustic features–for example, high-frequency bursts of wide-band noise are important for fricatives, while glides and liquids do not contain these but instead rely on changing frequency relations between more discrete tonal energy bands called formants [10]. Any attempt to compare acoustic similarity among consonants that do not contain the same structural acoustic features runs up against the unsolved problem of how to weight differences in shared and unshared features in a perceptually meaningful way. Consonants also depend on dynamically-varying acoustic properties which are more difficult to measure robustly. Vowels, on the other hand, are an important class of speech sounds that all have a similar acoustic structure, allowing a subset of perceptually-relevant acoustic features to be measured and compared in a robust and uniform way. Physical acoustic distances for each pair of vowels were defined by the differences in center frequencies of the three lowest energy bands or formants in their Discrete Fourier Transform spectrum, measured in the steady-state portion at the center of each vowel. By looking in detail at the correspondence between discrimination errors, phonological identification results and acoustic physical distance measures for the vowel portions of the stimuli, the relative roles of processing based on phonology and processing based on physical acoustic similarity could be quantitatively evaluated.

If rotated vowel sounds are not initially processed phonologically, naïve discrimination errors should be inversely related to physical acoustic distance; any relations between phonological distance and discrimination errors should be due to phonological distances mirroring acoustic distances. Under this model, acoustic distances will significantly explain some proportion of the variance in discrimination performance, and phonological distances will not explain any significant variance in addition to that explained by acoustic distance. If, on the other hand, the sounds are initially processed by the native language phonological system, there should be an independent effect of phonological distance on the discrimination of such sounds–phonological distances should explain a significant proportion of the variance in discrimination performance in addition to that explained by acoustic distances.

## Results

### Discrimination performance

The naïve perception of rotated speech syllables was evaluated using a “same or different” discrimination experiment with a set of 20 naïve participants, none of whom reported perceiving any of the rotated syllables as speech. Subjects listened to trials containing three stimuli in a row, and judged whether the third stimulus was the same as the first or second stimulus or different from both. Separate runs tested discrimination of rotated consonant and vowel pairs, each using 10 subjects; the stimuli within each trial of a single run differed only by a consonant or a vowel respectively (see Materials and Methods). The number of erroneous “same” judgments over all the repetitions of each pair in different consonantal or vowel contexts was used to construct vowel and consonant discrimination error matrices for each subject, in which each cell represents the number of discrimination errors for the pair of vowels or consonants in the corresponding row and column (10 pairs from 5 vowels, 105 pairs from 15 consonants). These data averaged across subjects provided the matrix of discrimination errors for each vowel or consonant pair. Significant heterogeneity was found in the proportion of discrimination errors among different rotated vowel pairs, and among different rotated consonant pairs (Friedman nonparametric two-way ANOVA [11], F_r_ = 59.9, df = 9, p<0.0001 for vowels; F_r_ = 221, df = 104, p<0.0001 for consonants), demonstrating that rotated syllables initially have differential perceptual identities.

### Phonological identification

To assess whether phonology could play a role in the initial perceptual status of rotated sounds, we needed to assess the naive perceptual identity of rotated syllables when processed phonologically. An independent group of ten native-speaking Italian participants, who were naïve to rotated speech and to the purpose of the experiment, described their phonological perception of the rotated syllables by typing them onomatopoetically on a computer keyboard. The participants were told to describe each sound with a syllable, as would be used to explain what it sounded like to a friend who is not present, and were free to listen to each sound as many times as they liked before recording a response. All 10 participants reported that they did not perceive any of the rotated syllables as speech. Participants requested to hear each stimulus an average of 4.03±0.96 times before providing a description (ranging from 2.60±1.07 times to 7.10±3.69 for particular stimuli), indicating that the stimuli were not easy to consciously identify with speech sounds. The phonological identity of each rotated vowel and consonant was obtained from these descriptions. The phonological identities were averaged over all the repetitions of consonants and vowels in different contexts, and identification matrices were constructed for each subject. The close forms for the vowel ‘e’ and the vowel ‘o’ were combined into the same category as their respective open forms, since these were not separable by written symbols. Identification matrices for consonants and vowels showed high similarities among the individual participants, and were combined to produce summary identification matrices of the onomatopoetic responses of all participants (Tables 1 & 2 for vowels and consonants, respectively; additional information on the relatively rare identifications of rotated consonants as vowels is given in the Supporting Information Text S1, Tables S5, S6). Phonological distances between pairs of rotated vowels and consonants (5 vowels yield 10 paired comparisons, 15 consonants yield 105 paired comparisons) were obtained from these phonological descriptions by calculating the differences between the perceptual identification percentages of each pair of rotated vowels and consonants (see Materials and Methods).

**Table 1:** Percentages of identification of rotated vowels (rows) as unrotated vowels (columns).

	A	E	I	O	U
**A_R_**	20	64.3	1.3	4.7	9.7
**E_R_**	19.3	6.5	0	55	19.2
**I_R_**	0.7	1.4	8.8	64.3	24.8
**O_R_**	2.7	78.7	14.7	1.2	2.7
**U_R_**	0.7	20	78	0.7	0.6

**Table 2:** Percentages of identification of rotated consonants (rows) as unrotated speech sounds (columns).

	B	P	T	D	K	G	M	N	F	V	J	CH	L	R	S	Z	GN	GL	Vowel*	O/I§
**B_R_**	4	2	4	22	0	2	26	8	2	0	0	0	4	0	0	0	1	0	10	15
**P_R_**	10	16	12	12	0	4	4	0	0	0	0	0	0	2	0	0	0	0	8	32
**T_R_**	16	26	24	32	0	0	0	0	0	0	0	0	0	2	0	0	0	0	0	0
**D_R_**	14	0	0	56	0	0	2	8	0	0	0	0	2	0	18	0	0	0	0	0
**K_R_**	6	6	8	10	6	2	10	8	2	0	2	0	2	6	0	0	0	0	6	26
**G_R_**	14	2	0	12	0	6	18	10	2	0	0	0	4	6	10	0	0	0	4	12
**M_R_**	0	0	0	6	0	2	4	36	2	0	0	0	14	8	4	0	3	0	12	9
**N_R_**	0	0	0	2	0	8	0	22	0	0	2	0	24	12	4	6	0	3	8	9
**F_R_**	0	0	2	6	0	0	10	4	30	0	2	0	2	2	6	0	0	0	6	30
**V_R_**	2	4	2	18	0	4	16	18	4	0	0	0	4	0	10	2	0	0	4	12
**J_R_**	44	4	2	10	2	0	4	2	2	0	0	0	0	0	22	4	0	0	4	0
**CH_R_**	4	18	10	10	4	2	2	10	4	0	0	0	2	20	4	0	1	1	6	2
**L_R_**	4	0	0	12	0	0	0	18	0	2	0	0	20	18	4	16	0	0	4	2
**R_R_**	4	2	4	6	0	0	2	20	0	0	0	0	4	32	18	2	0	0	6	0
**S_R_**	0	0	4	18	0	0	2	8	14	0	0	0	6	10	26	0	0	0	2	10

* Vowel identity information provided in Supporting Information.

§ O/I = Omitted/Identical. Consonant was either omitted or given the same identity as the following vowel

**Figure 2:**
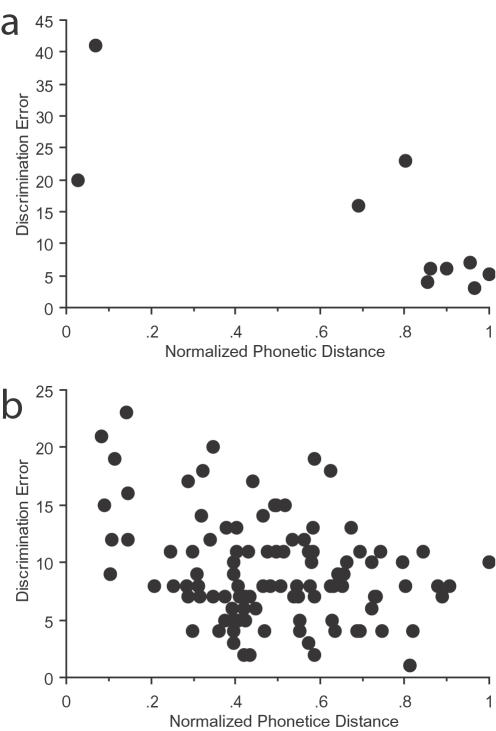
Discrimination error (average number of errors) as a function of phonological distance (normalized units) for (a) pairs of rotated vowels and (b) pairs of rotated consonants. In the vowel diagram (a), each point represents the average number of errors made over all subjects for the 15 repetitions involving each of the 10 vowel pairs (one repetition for each of the 15 consonantal contexts). In the consonant diagram (b), each point represents the average number of errors made over all subjects for the 5 repetitions involving each of the 105 consonant pairs (one repetition for each of the 5 vowel contexts).

The pattern of estimated phonological distances of rotated sounds were significantly related to the pattern of discrimination errors for both the rotated vowel and consonant pairs (Mantel test [12], Vowels r = −0.81, n = 10, p<0.008; Consonants r = −0.30, n = 105, p<0.002). Fewer discrimination errors were produced as phonological distances increased (Figure 2a for vowels, 2b for consonants), suggesting a significant relationship between naïvely-perceived phonological attributes of rotated stimuli and non-linguistic perceptual judgments about them. However, this does not address the potential role played by physical acoustic distance in these discrimination judgments, since phonologically similar sounds are also acoustically similar.

### Acoustic analysis

**Figure 3:**
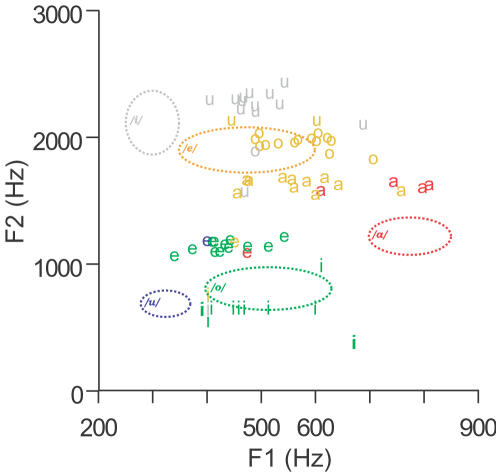
Second formant frequency (F2, y-axis) as a function of first formant frequency (F1, x-axis) for spectrally-rotated vowels. The letters used as plotting symbols denote the identity of each stimulus vowel before rotation (a = */a/*, e = */e/*, i = */i/*, o =  */o/*, u = */u/*). The dotted lines outline the elliptical centers of Italian vowel categories [13] and are labeled with their corresponding phonetic identities; the color of each ellipse codes vowel identity and is used to indicate the most common percept of each vowel stimulus (the color of its plotting symbol).

To analyze the role that physical acoustic similarities play in naïve rotated speech perception, we focused on an important subset of the information in the stimulus sounds, the vowels, for reasons explained in the introduction. The perception of vowel sounds has previously been shown to be related to the frequencies of the first two or three formants (resonant bands of energy concentration) during phoneme release [13]. Figure 3 shows rotated vowel stimuli plotted according to their first and second format values, together with ellipses indicating the natural borders of the five cardinal Italian vowel categories used in this experiment [14] (open and close forms of ‘e’ and of ‘o’ merged). Each rotated vowel in Figure 3 is also labeled according to the highest percentage of the natural vowel category used to phonologically identify it. The spectral rotation center frequency used in this experiment had a special effect on the vowel /i/, leaving it with two widely-spaced formants (at ∼500 Hz and 2.8 KHz) which listeners described as being perceptually segregated into a single formant at ∼500 Hz and a high frequency tone. Previous work [15] has shown that vowels with a single identifiable formant are perceived according to the vowel whose average of first and second formant values the single formant is closest to; the analysis of the rotated vowel /i/ was carried out accordingly (the same pattern of results reported below is found if all items containing the vowel /i/ are omitted). Acoustic similarities for rotated vowel pairs were defined by the difference in center frequency of the three lowest energy bands (formants) in the steady-state portion at the center of each vowel (sounds with smaller frequency differences between their formants have smaller distances and are more similar), as explained in the Materials and Methods. The distance between each rotated vowel and the center of each natural vowel category was similarly calculated, and the distances were averaged over the repetitions of each vowel in different consonantal contexts to construct a matrix representing the distances between each rotated vowel category and each natural vowel category. The identification matrix for rotated vowels had a high degree of correspondence with the matrix of rotated vowel category distances from natural vowel category centers (Spearman rank-order correlation coefficient [11], rho = −0.80, n = 25, p<0.0001), with rotated vowels being identified with the acoustically-closest natural vowel category, indicating that the naïve perceptual identity of rotated vowels closely follows the formant values used in the native vowel classification scheme [13], [14].

### Factors influencing discrimination performance

**Figure 4:**
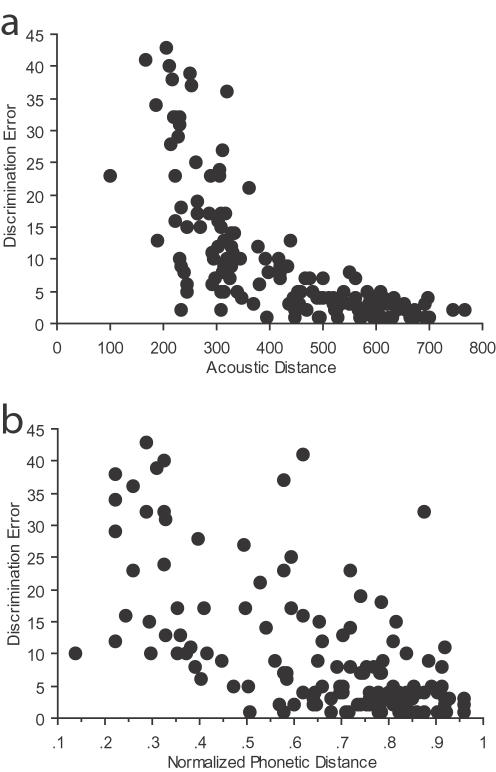
Discrimination error versus (a) acoustic distance (in Hz) and (b) phonological distance (normalized units) for vowels. Each data point in each diagram represents the average number of errors made over all subjects for each vowel pair discrimination in each consonantal context (10 vowel pairs×15 consonantal contexts = 150 data points). The data shown in (b) is represented in a different format in Figure 2b, where all of the 15 consonantal contexts involving the same vowel pair discrimination have been combined (for a total of 10 data points).

The relation between acoustic distances of the vowel pairs and discrimination errors (10 vowel pairs repeated in 15 consonantal contexts, for a total of 150 pairs, Figure 4a), was significant (Mantel test, r = −0.82, n = 150, p<0.0001), with fewer discrimination errors produced as acoustic distances increased. The relationship between discrimination errors and phonological distances for the same 150 pairs (Figure 4b) was also significant (Mantel test, r = −0.63, n = 150, p<0.0001).

The joint relationship of phonological distance and acoustic distance to discrimination performance was examined via stepwise linear multivariate regression. Phonological (PD) and acoustic (AD) distances both contributed significantly toward explaining variation in discrimination errors (F = 112, df = 2, p<0.0001, using both forward and backward models: F-to-remove = 31.3 for PD; F-to-remove = 76.9 for AD). Partial correlation analyses revealed that PD was significantly correlated with discrimination errors when AD was controlled for (r = −0.42, n = 150, p<0.0001), and that discrimination errors and AD were significantly correlated when PD was controlled for (r = −0.59, n = 150, p<0.0001); PD and AD were not themselves significantly correlated when each of their correlations with discrimination errors were controlled for (r = −0.15, n = 150, p = 0.07). These analyses clearly demonstrate that acoustical and phonological properties independently and jointly influence the naive perception of rotated vowels within the context of rotated syllables, and that acoustic distance does not account for the relationship between phonological distance and discrimination errors. Thus, phonological properties play a significant role in the naïve perception of these subjectively “non-speech” sounds.

## Discussion

These results demonstrate that acoustic and phonological properties independently affect naive discrimination of rotated vowel stimuli when presented within the context of rotated syllables. The exponential form of the relationship between phonological and acoustic variation in Figure 4a suggests that when two rotated vowels are acoustically dissimilar, they will be discriminated well regardless of their phonological relationships; as the sounds become more acoustically similar, phonological properties play a greater role in discrimination performance. In the case of the rotated speech sounds studied here, the perceptual system appears to unconsciously utilize phonological information to disambiguate similar sounds, even when these sounds are judged to be non-speech-like.

Previous investigators have proposed separate but parallel perceptual processes for speech and non-speech sounds that “compete” for perceptual attention, with speech consciously pre-empting or dominating non-speech when phonological percepts are present [4], [16], [17]. Our results suggest that naively processing rotated syllables in a non-speech mode results in phonetic processing without conscious awareness of the phonetic nature of the stimuli. At a minimum, these results suggest a nuanced relationship between conscious percepts and unconscious modes of speech processing that needs to be re-examined in more detail. Neural imaging experiments using spectrally-rotated speech [1]–[3], [6], [7] and other manipulated or synthetic sounds [4], [5] as control stimuli for disambiguating brain mechanisms associated with phonological processing need to include rigorous behavioral evaluations of how such stimuli are actually perceived, using more extensive perceptual testing than has been carried out to date. The fact that we went to extra lengths to minimize the ease with which the stimuli used here could be naively perceived in a speech-like manner further underscores this issue. Perhaps the most important implication of this work is that conscious subjective judgments of perceptual “speechiness” should no longer be considered adequate grounds for categorizing sounds for experimental purposes, especially if brain activation patterns are going to be compared among classes of stimuli. Sounds not consciously perceived as speech may still be processed (at least partially) in a speech-like manner.

## Materials and Methods

### Stimuli

Stimuli were created from 75 CV syllables (5 vowels, considering the open and closed forms of ‘e’ and of ‘o’ as single vowel categories; 15 consonants [the consonants z, ts, dz, gn, gl, sh were not used in the stimuli]) recorded from a single male Italian speaker, who gave written informed consent for his participation in this experiment. The syllables were approximately 500 ms long with a steady state vowel portion occupying between 120–250 ms of their total length. Spectrally-rotated syllables (rotation frequency = 1.5 kHz) were created by applying a digital version of the algorithm introduced by Blesser [8], implemented in custom Matlab (The Mathworks, Natick, MA, USA) code, and applied to the recorded syllables. The algorithm consisted of three steps: (1) low pass filtering below 2.8 kHz; (2) modulation of the signal by a cosine wave at 3 kHz; (3) low-pass filtering the modulated sound below 2.8 kHz, resulting in a “mirror image” of the original spectrum around 1.5 kHz. The choice of this rotation frequency involved a compromise between preservation of maximum acoustic information and creation of a signal that would have a low a-priori probability of being initially heard in a speech-like manner. We evaluated the perceptual status of rotated speech with many rotation frequencies; the frequency employed here was the best compromise we could find between “non-speechiness” and the constraints imposed by low-pass filtering (to preserve speech information in the rotated waveform). This center frequency resulted in sounds that were judged by experienced and naïve listeners to be less speech-like in quality than higher rotation frequencies (see Supporting Information Text S1, Tables S1, S2, S3, S4). Thus, we deliberately attempted to make these sounds as dissimilar to speech as possible given the technical constraints of the spectral rotation technique.

For quantifying the acoustical properties of the rotated vowels, the first three formants of the rotated vowel stimuli were estimated. The formants of rotated vowels were obtained by transforming each formant of the original (unrotated) signal F_i_ to the value 3000-F_i_. For each unrotated vowel the formant values were measured in a 100 ms window in the steady state portion of the middle of the vowel using the LPC algorithm provided in the Praat software package [18]; this method was validated by checking the measured values against formant peaks observed directly from discrete Fourier transform spectra, and was found to be accurate.

### Experimental design

Ethics approval for these experiments was obtained from the SISSA Institutional Review Board; written informed consent was obtained from all participants.

A group of naïve, native Italian speakers participated in the discrimination experiment using spectrally-rotated CV syllable stimuli. The experiment was divided into two parts, testing vowel and consonant discrimination separately, each using ten subjects. A two-alternative forced choice ABX procedure (X = A or X = B) was used to examine consonants, and a three-alternative forced choice ABX procedure (X = A or X = B or X is neither A nor B) was used to examine vowels. The interstimulus intervals were 500 ms between A and B, and 800 ms between B and X. The three-alternative forced choice procedure was adopted for vowels based on the results of pilot experiments that found that the two-alternative procedure produced a small number of discrimination errors with vowels; the three-choice procedure was adopted to increase the number of errors and to avoid a ceiling effect. For the trials in which X was neither A nor B, errors were counted for the pair consisting of X and the A or B stimulus that X was incorrectly perceived as. Stimuli on each trial varied either only by a rotated vowel (in the vowel test) or only by a rotated consonant (in the consonant test); trials were repeated for all possible rotated vowel and consonant combinations. The subjects were instructed to listen to each sound sequence and to decide whether the final sound was identical to the first or second sound (consonant test), or identical to the first or second sound or neither (vowel test). Responses were indicated by pressing predefined computer keyboard keys. Subjects were kept blind to the linguistic origin of the stimuli, and to the purpose of the study before the experiment, and were instructed to respond as quickly as possible. The number of incorrectly discriminated trials for each vowel or consonant pair was used to calculate separate error matrices for vowels and consonants.

### Estimation of phonological and acoustic similarity

Ten native Italian speakers with no previous experience with rotated speech (none of whom had participated in the discrimination experiments) listened to a randomized list of rotated syllables and typed onomatopoetic versions of their perceptions on a computer keyboard. In Italian, there is a good correspondence between spoken and written forms. Participants were instructed to listen to each sound and type an Italian syllable that they would best use to describe that sound. They were free to listen to each syllable as many times as they liked before providing a response. Each subject provided one final judgment on each of the 75 stimuli (15 consonants for each of the five cardinal vowels, open and close forms of ‘e’ and of ‘o’ assigned to single categories). Their final judgments, and the number of times each participant listened to each stimulus, were recorded by a custom-written experimental control program. The phonetic percepts for each consonant and vowel were obtained from the typed responses to each syllable. Identification matrices for consonants and vowels were constructed from the results of each participant. Each row represented one rotated vowel (or consonant), and each column represented a natural vowel (or consonant) category, so that each cell represented the percentage of examples of a particular rotated vowel (or consonant) identified within a natural phonological category. Initial examination of the responses revealed a similar pattern across participants, so their responses were combined into a single identification matrix used for further analysis.

The phonological distances between each pair of rotated vowels or consonants were estimated from the identification matrix by measuring the RMS distance (RMS = root-mean-square) between the two rows of the matrix containing the identification data for the two sounds of the pair. Root-mean-square indicates the mathematical operations of squaring the differences between corresponding numbers in the two sets, taking the mean of these differences, and then taking the square root of this mean. If two rotated sounds have a profile of identification with natural sounds containing similar percentages, their RMS distances will approach zero; pairs of rotated sounds with different percentage identification profiles will have larger RMS distances which will depend on the degree of difference between their percentage identification profiles.

The acoustic distances between two rotated vowels with three formants (all vowels except ‘i’) were estimated from their Euclidean distances in a space defined by the center frequencies of their three lowest formants. When rotated around 1.5 kHz, the vowel ‘i’ had two widely-spaced formants that are perceptually segregated as a low frequency formant and a high frequency tone. Maurer et al [15] have demonstrated that a single formant vowel is perceived according to the vowel whose average of first and second formant values the single formant is closest to. The same procedure was adopted here to estimate the acoustic similarity between rotated ‘i’ vowels (with two frequency bands) and all other vowels (with three frequency bands). Non-i vowels were transformed into a two-frequency-band version for comparison with ‘i’-vowels by averaging their first two formants, and the Euclidean distance between the two-frequency-band versions was calculated. The pattern of the results in all analyses reported here is no different if all stimuli containing the vowel /i/ are omitted from the analyses.

### Statistical analyses

Linear multivariate regression, correlation and partial correlation analyses were run using Statview (SAS Institute, Cary NC) and MATLAB; Mantel tests were run using a MATLAB script based on the implementation described by Manly [12].

## Supporting Information

Text S1 (0.02 MB DOC)

Table S1 Percentages of identification of MANNER feature properties after rotation.
(0.03 MB DOC)

Table S2 Percentages of identification of PLACE feature properties after rotation.
(0.03 MB DOC)

Table S3 Percentages of identification of VOICING feature properties after rotation.
(0.03 MB DOC)

Table S4 Comparison of consonant identification performance with the Blesser study [s1].
(0.03 MB DOC)

Table S5 Percentages of identification of rotated consonants (rows) as unrotated vowels (columns) different from the following vowel in the syllable
(0.04 MB DOC)

Table S6 Relative percentage of rotated consonants with the indicated feature (rows) identified as a vowel (columns), and the relative mutual information between rotated consonant features and vowel identification (rMI).
(0.04 MB DOC)

Audio S1 Normal Syllables. The syllabic sequence \ba\-\be\-\bo\-\bu\ pronounced by a male speaker.
(0.22 MB WAV)

Audio S2 Spectrally-rotated Syllables. The spectrally-rotated syllabic sequence \ba\-\be\-bo\-\bu\ pronounced by a male speaker (rotation frequency = 1.5 kHz).
(0.22 MB WAV)
