# Supplementary material for: Phonological Representations Are Unconsciously Used when Processing Complex, Non-Speech Signals
Source: PLoS One. 2008 Apr 16;3(4):e1966. doi: 10.1371/journal.pone.0001966 (PMC2292097; doi:10.1371/journal.pone.0001966)
Supplement: Text S1 — (0.02 MB DOC) [file pone.0001966.s001.doc]

**Supporting Information**

**Consonant feature analysis**

To quantify how much of the phonological information about consonants was preserved after spectral rotation, the average consonant identification matrix (Table 2 in the paper) was analyzed according to three consonant features: manner of articulation, place of articulation and voicing [s1]. The subcategories for manner of articulation are Plosive (‘b’, ‘p’, ‘t’, ‘d’, ‘k’, ‘g’), Nasal (‘m’, ‘n’, ‘gn’, ‘gl’) or Fricative (‘f’, ‘v’, ‘j’, ‘ch’, ‘l,’ ‘r’, ‘s’, ‘z’, ‘ts’, ‘dz’); place of articulation subcategories consist of Front (‘b’, ‘p’, ‘m’, ‘f’, ‘v’), Middle (‘d’, ‘t’, ‘n’, ‘s’, ‘z’, ‘ts’, ‘dz’) or Back (‘k’, ‘g’, ‘ch’, ‘j’, ‘gn’, ‘gl’); and voicing subcategories include Voiced (‘b’, ‘d’, ‘g’, ‘m’, ‘n’, ‘v’, ‘j’, ‘l’, ‘r’, ‘z’, ‘dz’) or Unvoiced (‘p’, ‘t’, ‘k’, ‘f’, ‘ch’, ‘s’, ‘ts’).

The feature identification matrices (Table S1, S2, S3) represent the percentage of time that rotated consonants were perceived to have the same (diagonal) or different (off-diagonal) feature properties than the unrotated consonant on which they were based. The “mean same” column represents the average of the diagonal entries for the matrix. The comparison of these values (rotation frequency 1.5 kHz) with those that Blesser [s1] measured in naïve subjects (rotation frequency 1.6 kHz) is given in Table S4. The information about consonant features was more preserved in Blesser’s study (1.6 kHz rotation frequency).

**Consonants perceived as vowels**

From 0-13 % of the time (median 4 %), participants in the phonetic identification experiment heard rotated consonants as vowels different from the ones that followed the consonant (Table S5). When these are arranged according to features such as place of articulation, manner of articulation, and voicing, a pattern emerges, which is made clearer by converting the values to percentages relative to the total number of stimuli for each consonant heard as a vowel (Table S6). Relative mutual information (Miller and Nicely 1955, [s3]), a measure of the degree to which rotated consonant features co-vary with vowel identity (minimum possible = 0 and maximum possible = 1), is also shown. Vowels that rotated consonants have been identified as are better predicted by the place of articulation properties of the consonant (front, middle or back) than by manner or voicing properties. Place contrasts in consonants are given by such acoustic cues as the second formant value and the direction of its transitions to the following vowel [s2], which appear in the rotated case to contribute toward the identity of the vowel the consonant is going to be perceived as.

**Supporting Information Methods**

The relative Mutual Information (information transmission) for the matrix representing identification percentage of each consonant feature by natural vowels was calculated by the method explained in [s3]. If the row variable (stimulus) x assumes discrete values i=1,2,…,k each with probability p_i_, and the column variable (response) y assumes discrete values j=1,2, …,m each with probability p_j_, the relative mutual information (rMI) between the stimuli and the responses is given by:

$rMI(x;y)=\frac{-\sum_{i,j} p_{ij}\log(\frac{p_{i}p_{j}}{p_{ij}})}{-\sum_{i} p_{i}\log(p_{i})}$

rMI(x,y) is the transmission from x to y in bits per stimulus. If transmission is poor and the response is not closely correlated to the stimulus, rMI(x,y) will be close to zero; it approaches its maximum value when the stimulus can be completely predicted from the response. The frequencies of stimuli and responses were obtained directly from the identification matrix containing the percentage occurrences of each consonant feature (as stimulus) and each natural vowel (as response), shown in Table S6.

**Supporting Information References**

s1. Blesser B. (1972) Speech perception under conditions of spectral transformation. J Speech Hear Res 15: 5-41.

s2. Wright, R (2004) A review of perceptual cues and cue robustness. In: Hayes B, Kircher RM, Steriade D, editors. Phonetically Based Phonology. Cambridge University Press, Cambridge. pp. 34-57.

s3. Miller, GA and Nicely, PE (1955) An analysis of perceptual confusions among some English consonants. J Acoust Soc Amer 27, 338-352.
