# Supplementary material for: Phonological Representations Are Unconsciously Used when Processing Complex, Non-Speech Signals
Source: PLoS One. 2008 Apr 16;3(4):e1966. doi: 10.1371/journal.pone.0001966 (PMC2292097; doi:10.1371/journal.pone.0001966)
Supplement: Table S1 — Percentages of identification of MANNER feature properties after rotation. (0.03 MB DOC) [file pone.0001966.s002.doc]

**Table S1. Percentages of identification of MANNER feature properties after rotation.**

| **MANNER** |  |  |  |  |
| --- | --- | --- | --- | --- |
|  | *Plosive* | *Nasal* | *Fricative* | *Mean Same* |
| *Plosive_R_* | 65.9 | 20.5 | 13.5 | 49.6 |
| *Nasal_R_* | 14.9 | 35.7 | 94.4 |  |
| *Fricative_R_* | 33.5 | 19.1 | 47.3 |  |

Diagonal values represent the percentage of identifications that had the same manner as the unrotated consonant that the stimulus was based upon.
