# Supplementary material for: Phonological Representations Are Unconsciously Used when Processing Complex, Non-Speech Signals
Source: PLoS One. 2008 Apr 16;3(4):e1966. doi: 10.1371/journal.pone.0001966 (PMC2292097; doi:10.1371/journal.pone.0001966)
Supplement: Table S2 — Percentages of identification of PLACE feature properties after rotation. (0.03 MB DOC) [file pone.0001966.s003.doc]

**Table S2. Percentages of identification of PLACE feature properties after rotation.**

| **PLACE** |  |  |  |  |
| --- | --- | --- | --- | --- |
|  | *Front* | *Middle* | *Back* | *Mean Same* |
| *Front_R_* | 38.9 | 56 | 5.2 | 43.1 |
| *Middle_R_* | 15.8 | 82.4 | 1.8 |  |
| *Back_R_* | 38.2 | 53.7 | 8 |  |

Diagonal values represent the percentage of identifications that had the same place as the unrotated consonant that the stimulus was based upon.
