# Supplementary material for: Phonological Representations Are Unconsciously Used when Processing Complex, Non-Speech Signals
Source: PLoS One. 2008 Apr 16;3(4):e1966. doi: 10.1371/journal.pone.0001966 (PMC2292097; doi:10.1371/journal.pone.0001966)
Supplement: Table S3 — Percentages of identification of VOICING feature properties after rotation. (0.03 MB DOC) [file pone.0001966.s004.doc]

**Table S3. Percentages of identification of VOICING feature properties after rotation.**

| **VOICING** |  |  |  |
| --- | --- | --- | --- |
|  | *Voiced* | *Unvoiced* | *Mean Same* |
| *Voiced_R_* | 77.9 | 11.9 | 56.4 |
| *Unvoiced_R_* | 47.3 | 35 |  |

Diagonal values represent the percentage of identifications

that had the same voicing as the unrotated consonant that

the stimulus was based upon.
