# Supplementary material for: Phonological Representations Are Unconsciously Used when Processing Complex, Non-Speech Signals
Source: PLoS One. 2008 Apr 16;3(4):e1966. doi: 10.1371/journal.pone.0001966 (PMC2292097; doi:10.1371/journal.pone.0001966)
Supplement: Table S4 — Comparison of consonant identification performance with the Blesser study [s1]. (0.03 MB DOC) [file pone.0001966.s005.doc]

**Table S4. Comparison of consonant identification performance with**

**the Blesser study [s1]**

|  | **Present data** | **Blesser data** |
| --- | --- | --- |
| **Manner** | 49.6 | 78.7 |
| **Place** | 43.1 | 36.3 |
| **Voicing** | 56.4 | 87.4 |
| ***Average*** | ***49.7*** | ***67.5*** |
