# Supplementary material for: Phonological Representations Are Unconsciously Used when Processing Complex, Non-Speech Signals
Source: PLoS One. 2008 Apr 16;3(4):e1966. doi: 10.1371/journal.pone.0001966 (PMC2292097; doi:10.1371/journal.pone.0001966)
Supplement: Table S5 — Percentages of identification of rotated consonants (rows) as unrotated vowels (columns) different from the following vowel in the syllable (0.04 MB DOC) [file pone.0001966.s006.doc]

**Table S5. Percentages of identification of rotated**

**consonants (rows) as unrotated vowels (columns)**

**different from the following vowel in the syllable**

|  | **A** | **E** | **I** | **O** | **U** |
| --- | --- | --- | --- | --- | --- |
| **B_R_** | 2.0 | 6.0 | 0.0 | 2.0 | 0.0 |
| **P_R_** | 0.0 | 5.7 | 0.0 | 1.9 | 0.0 |
| **T_R_** | 0.0 | 0.0 | 0.0 | 0.0 | 0.0 |
| **D_R_** | 0.0 | 0.0 | 0.0 | 0.0 | 0.0 |
| **K_R_** | 0.0 | 1.9 | 0.0 | 1.9 | 1.9 |
| **G_R_** | 2.0 | 0.0 | 0.0 | 0.0 | 2.0 |
| **M_R_** | 9.8 | 2.0 | 0.0 | 0.0 | 0.0 |
| **N_R_** | 3.9 | 0.0 | 0.0 | 3.9 | 0.0 |
| **F_R_** | 0.0 | 5.5 | 0.0 | 0.0 | 0.0 |
| **V_R_** | 0.0 | 1.9 | 0.0 | 1.9 | 0.0 |
| **J_R_** | 0.0 | 0.0 | 0.0 | 0.0 | 4.0 |
| **CH_R_** | 0.0 | 0.0 | 0.0 | 0.0 | 5.9 |
| **L_R_** | 3.9 | 0.0 | 0.0 | 0.0 | 0.0 |
| **R_R_** | 5.8 | 0.0 | 0.0 | 0.0 | 0.0 |
| **S_R_** | 2.0 | 0.0 | 0.0 | 0.0 | 0.0 |
