# Supplementary material for: Phonological Representations Are Unconsciously Used when Processing Complex, Non-Speech Signals
Source: PLoS One. 2008 Apr 16;3(4):e1966. doi: 10.1371/journal.pone.0001966 (PMC2292097; doi:10.1371/journal.pone.0001966)
Supplement: Table S6 — Relative percentage of rotated consonants with the indicated feature (rows) identified as a vowel (columns), and the relative mutual information between rotated consonant features and vowel identification (rMI). (0.04 MB DOC) [file pone.0001966.s007.doc]

**Table S6. Relative percentage of rotated consonants with the indicated feature (rows) identified as a vowel (columns), and the relative mutual information between rotated consonant features and vowel identification (rMI)**

|  | **A** | **E** | **I** | **O** | **U** | **rMI** |
| --- | --- | --- | --- | --- | --- | --- |
| **MANNER** |  |  |  |  |  |  |
| *Plosive_R_* | 14.7 | 49.8 | 0.0 | 21.2 | 14.3 | 0.190 |
| *Nasal_R_* | 69.9 | 10.2 | 0.0 | 19.9 | 0.0 |  |
| *Fricative_R_* | 37.9 | 23.9 | 0.0 | 6.1 | 32.0 |  |
| **PLACE** |  |  |  |  |  |  |
| *Front_R_* | 30.6 | 54.4 | 0.0 | 15.0 | 0.0 | 0.470 |
| *Middle_R_* | 79.9 | 0.0 | 0.0 | 20.1 | 0.0 |  |
| *Back_R_* | 10.1 | 9.7 | 0.0 | 9.7 | 70.5 |  |
| **VOICING** |  |  |  |  |  |  |
| *Voiced_R_* | 53.6 | 19.4 | 0.0 | 15.4 | 11.7 | 0.220 |
| *Unvoiced_R_* | 7.5 | 49.0 | 0.0 | 14.2 | 29.3 |  |
